# Prevalence and associated factors of ischemic heart disease (IHD) among patients with diabetes mellitus: a nation-wide, cross-sectional survey

**DOI:** 10.1186/s12872-018-0887-0

**Published:** 2018-07-27

**Authors:** Boonsub Sakboonyarat, Ram Rangsin

**Affiliations:** Department of Military and Community Medicine, Phramongkutklao College of Medicine, Bangkok, 10400 Thailand

**Keywords:** Ischemic heart disease, Diabetes mellitus, Nation-wide survey, Prevalence, Associated factors

## Abstract

**Background:**

Ischemic Heart Disease (IHD) is the first ranked among most common causes of death involving cardiovascular and other diseases. The information on the prevalence of IHD in Thailand is lacking especially among patients with diabetes mellitus. The objectives of this study were to determine the prevalence of IHD among patients with diabetes mellitus and to determine factors associated with IHD in a nation-wide survey.

**Methods:**

A cross-sectional study to assess national outcomes among patients with diabetes who visited 831 public hospitals in Thailand was conducted in 2013 to evaluate status of care among patients with diabetes aged at least 18 years who received medical treatment in the target hospital for the last 12 months.

**Results:**

A total of 25,902 patients with diabetes were included in this study. IHD was detected among 918 patients (3.54%; 95%CI 3.32–3.77). Multivariate analysis was conducted to determine which factors were most associated with IHD, and the results showed age (AORs 1.05; 95%CI 1.04–1.05), being male (AORs 1.78; 95%CI 1.53–2.07), hypertensive comorbidity (AORs 2.10; 95%CI 1.68–2.62), being in Health Region 4 (AORs 1.93; 95%CI 1.54–2.35), presenting hyperglycemic crisis (AORs 1.53; 95%CI 1.14–2.06) and insulin therapy (AORs 1.40; 95%CI 1.17–1.66) were the highest associated factors for IHD in this population.

**Conclusion:**

Our data emphasized that IHD was a problem among patients with diabetes. Diabetic patients should be regularly assessed for IHD and their risk factors should be better controlled. Moreover, the Ministry of Public Health managers and clinicians should provide further preventative strategies to attenuate cardiovascular disease.

**Electronic supplementary material:**

The online version of this article (10.1186/s12872-018-0887-0) contains supplementary material, which is available to authorized users.

## Background

Ischemic Heart Disease (IHD) is the first ranked and most common cause of death in cardiovascular and overall diseases [1]. The estimated prevalence of IHD among people aged ≥18 years in 2013 was 6.1, 6.4, 5.3 and 3.7% in Caucasian, African, Latino and Asian populations, respectively [1]. The prevalence increased with age and more prevalence was noted among males [2]. One recent study showed that the number of estimated deaths caused by IHD in Southeast Asia increased from 5.73 to 8.14 million from 1990 to 2013 [3]. Prevalence of IHD increased in patients with potential risk factors such as diabetes mellitus [4, 5]. Globally, adults with diabetes total 381 million. The estimated global prevalence of diabetes mellitus among adult populations was 8.3, 9.6, 5.7, 6.8, 8.6 and 6.4% in North American and Caribbean (NAC), African, European, Western Pacific and Thai populations [6]. Diabetes mellitus increases independent risk of IHD approximately 1.5 and 1.7 fold among males and females, respectively [7]. In 2007, a related study conducted among patients with diabetes reported that the age-standardized incidence rate (per 1000 person-years) of first coronary heart disease (CHD) events was 28.8 among males and 23.3 among females [8].

Epidemiological data of IHD in Asian populations has been studied in many countries. From 2007 to 2008, the prevalence of CHD in China was 0.63% [9]. In 2002, the overall prevalence of CHD in India was 8.2% [10]. However, limited information is available regarding the prevalence of IHD in the Thai general population in 1991 was 0.99% [11], especially among patients with major risk factors such as diabetes mellitus. We determined the prevalence of IHD among patients with diabetes mellitus using a nation-wide, cross-sectional survey among patients with diabetes mellitus. The secondary objective of this study was to determine factors associated with IHD.

## Methods

### Study designs

The data of this study were retrieved from database: An assessment in Quality of Care among Patients Diagnosed with Type2 Diabetes and Hypertension Visiting Ministry of Public Health and Bangkok Metropolitan Administration Hospital in Thailand (Thailand DM/HT) after the permission of the Medical Research Network of the Consortium of Thai Medical Schools (MedResNet).

The Thailand DM/HT evaluation survey was a nation-wide, cross-sectional study aiming to assess outcomes among patients with diabetes visiting public hospitals of the Ministry of Public Health (MoPH), Thailand and private hospitals and clinics in Bangkok was conducted from 2012 to 2013. The main objective of the Thailand DM/HT evaluation survey was to evaluate the status of care and was supported by the Thai National Health Security Office (NHSO).

### Subjects

The healthcare system in Thailand can be categorized in two types comprising (1) healthcare under the MoPH and (2) private healthcare such as private clinics and hospitals. All Thais have healthcare coverage schemes such as the universal coverage scheme, social insurance scheme and government officer scheme. These healthcare schemes are supported by the NHSO. All of the hospitals under the MoPH at all levels, i.e., community (district), general (provincial) and regional nationwide and some private clinics in Bangkok were invited to participate in the study.

A stratified two-stage cluster sampling method proportional to the size was used to select national and provincial representative samples of patients with diabetes in Thailand. The stratified sample was drawn from a subset of all MoPH hospitals in Thailand. For Bangkok, the targeted institutes included all hospitals and clinics under the NHSO. The first level (province) comprised 77 strata while the second level constituted hospitals within each province. The second level was categorized in 5 strata by size, i.e., regional center hospital (> 500 beds), provincial general (middle) hospital (200–500 beds), first level one (F1) (90–120 beds), first level two (F2) hospital (60 beds) and first level three (F3) hospital (10–30 beds). All university medical centers were excluded from this study.

Inclusion criteria for this study comprised patients with diabetes aged at least 18 years receiving hospital medical treatment in hospital, drawn using the specified sampling method, during the previous 12 months. Any patient who had participated in a clinical trial was excluded. Those patients may have received trial medication or placebo, influencing the outcome of the study.

### Data collection

A total of 833 hospitals under the MoPH were categorized as 33 regional hospitals, 83 general hospitals and 717 first level or community hospitals including the first level one (F1) 73 hospitals, the first level two (F2) 126 hospitals and the first level three (F3) 518 hospitals. All regional and general hospitals were selected, as well as 10% of F1 hospitals, 20% of F3 hospitals and 70% of F3 hospitals. This faction was based on the proportion of patient care provided at the various levels of hospitals. Patients with a diagnosis of diabetes mellitus were randomized and registered at each hospital. A standardized case report form was used to obtain the required information from medical records and was sent to the Thailand DM/HT study of the Medical Research Network of the Consortium of Thai Medical Schools (MedResNet) central data management unit in Nonthaburi. Data were retrieved from patient’s medical records, status of diabetes complications and results of laboratory tests.

### Measurements

Data collected included demographics, weight, height, body mass index (BMI), waist circumference, smoking behavior, systolic blood pressure (SBP), diastolic blood pressure (DBP), cardiovascular complications such as left ventricular hypertrophy (LVH), diabetic complications such as diabetic retinopathy (DR) and diabetic nephropathy (DN), blood chemistry data including fasting plasma glucose (FPG), hemoglobin A1c (HbA1c), hematocrit (Hct), hemoglobin (Hb), serum creatinine (Cr), uric acid, lipid profile including total cholesterol (TC), triglyceride (TG), high density lipoprotein cholesterol (HDL) and low density lipoprotein cholesterol (LDL), available electrocardiogram (ECG) data and results, history of anti-hyperglycemic and antiplatelet drug use and glomerular filtration rate (GFR) calculated using the epidemiology collaboration formula (EPI). Our study enrolled those patients with diabetes who were diagnosed and received ongoing medical care in a hospital. Hospitals in Thailand normally use the standard diagnosis and treatment following Thai clinical practice guidelines for diabetes and diagnosis and classification of diabetes mellitus using Diabetes Care, 2010. Diabetes mellitus was defined as FPG ≥126 mg/dl and confirmed by repeat testing at a second visit. Fasting is defined as no caloric intake for at least 8 h [12]. IHD was defined as myocardial infraction or history of coronary revascularization. Diagnosis of myocardial infraction was performed using standard criteria including stable angina and acute coronary syndrome, categorized as ST-T segment elevation myocardial infraction, nonST-T segment elevation myocardial infraction and unstable angina.

### Statistical analysis

Data were coded and entered in the STATA/MP for Windows, Version 12 (Stata Corp LP, TX). Categorical data were presented as number and percentage. Continuous data were presented as mean and standard deviation (SD). Prevalence was analyzed using descriptive statistics and reported as percentage and 95% confident interval. The chi-square test was used to compare categorical data. Continuous data was compared using the *t*-test. Continuous data were grouped to analyze associated factor using odds ratio (OR). The magnitude of associations was presented as crude ORs with 95% confidence interval. The multivariate analysis was performed using logistic regression analysis and Forward Stepwise (LR) to adjust confounders. Stepwise *p*-value for entry was 0.05, and *p*-value for removal was set at 0.10. The Hosmer-Lemeshow goodness-of-fit of the logistic regression models was performed with *p*-value = 0.494. The complete case analysis and imputation method were used for any missing data. Missing values were imputed based on the means of the complete case. A *p*-value less than 0.05 was considered statistically significant.

## Results

### Demographic data

**Fig. 1:**
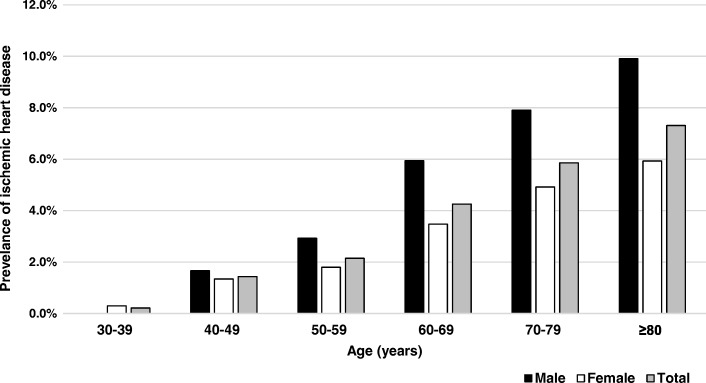
Prevalence of ischemic heart disease in male and female at different age groups

A total of 25,902 patients with diabetes mellitus were enrolled in this study from 2012 to 2013. Average age was 60.6 ± 10.5 years, 8076 (32.2%) were male and 17,836 (68.8%) were female. The average diabetes duration of patients was 7.1 ± 4.7 years while the average HbA1c level was 8.0 ± 2.2%. Baseline characteristics of this study are shown in Table 1. One third of participants lived in northeastern Thailand. In all, 62.5% subjects visited community or first level (F1, F2 and F3) hospitals. In this study, patients with hypertension totaled 29.1%. Prevalence of IHD among patients with diabetes was 3.54% (95%CI 3.32–3.77), increased with older age and was more common among males. Fig. 1 shows a bar graph representing IHD prevalence for every 10 years of age separated in male, female and overall.

**Table 1:** Baseline characteristics of patients (total number of subjects =25,902)

Baseline Variables	*n*	Mean ± SD or number (%)
Gender	25,902	
Female		17,836 (68.8)
Male		8076 (32.2)
Age	25,902	60.6 ± 10.5
Hospital level	25,902	
Regional center		3096 (12)
General provincial		4911 (19)
Community		16,187 (62.5)
Bangkok metropolitan administration		1708 (6.5)
Diabetic duration	25,902	7.1 ± 4.7
Waist circumference	17,488	88.5 ± 10.4
Body mass index (kg/m^2^)	24,643	25.5 ± 4.4
Fasting plasma glucose	23,048	154.7 ± 58.4
HbA1c level	20,481	8 ± 2.2
GFR_EPI	15,986	67.2 ± 31
LDL	15,544	108.6 ± 36.8
HDL	14,126	46.9 ± 13.8
Triglyceride	15,548	175.4 ± 110.9
Total cholesterol	14,775	187.7 ± 44.5
Uric acid	4553	6.1 ± 1.8
Smoking	23,806	
Never		21,249 (89.2)
Current Smoker		1036 (4.4)
Ex-smoker		1521 (6.4)
Hypertensive comorbidity	25,902	
No		7527 (29.1)
Yes		18,375 (70.9)

*SD* standard deviation, *HbA1c* hemoglobinA1c, *GFR_EPI* glomerular infiltration rate calculated by epidemiology collaboration formula, *LDL* low density lipoprotein cholesterol, *HDL* high density lipoprotein cholesterol

### Associated factors of IHD among patients with diabetes

Additional file 1 shows univariate analysis results regarding factors associated with IHD. Significant associated factors included older age, being male, health region, hospital level, insurance scheme, religion, occupation, GFR, HT comorbidity, HT duration, LVH, LAE, AF, DM duration, DN, DR and insulin, sulfonylurea, thiazolidinedione and aspirin therapy. Because a number of patients had missing values for HbA1c, this may have decreased the statistical power of the complete cases analysis. As a result, we applied imputation to handle the missing value and the results of multivariate analysis after imputation showed similarity to the original independent associations with IHD. Multivariate analysis showed the risk factors of IHD included age, being male, being in Health Region 4, hypertensive comorbidity and insulin therapy (Table 2).

**Table 2:** Multivariate logistic regression for factors associated with ischemic heart disease in diabetic patients. (*N* = 25,902)

Factors	IHD	No-IHD	AORs	95% CI
	*n* (%)	*n* (%)		
Age (years)	65.9 ± 9.5	60.4 ± 10.5	1.05^b^	(1.04–1.05)
Gender				
Female	523 (3)	17,303 (97)	1	
Male	395 (4.9)	7681 (95.1)	1.78^b^	(1.53–2.07)
Health regions				
Other health regions	774 (3.3)	22,877 (96.7)	1	
The 4th health region	144 (6.4)	2107 (93.6)	1.93^b^	(1.54–2.35)
Hypertensive Comorbidity				
No	129 (1.8)	7398 (98.2)	1	
Yes	789 (4.3)	17,586 (95.7)	2.10^b^	(1.68–2.62)
Hyperglycemic crisis				
No	846 (3.4)	23,680 (96.6)	1	
Yes	72 (5.2)	1304 (94.8)	1.53^a^	(1.14–2.06)
Insulin therapy				
No	659 (3.3)	19,685 (96.7)	1	
Yes	259 (4.7)	5299 (95.3)	1.40^b^	(1.17–1.66)

^a^*p* < 0.01, ^b^*p* < 0.001

*AORs* Adjusted odds ratio for age, gender, health regions, hypertensive comorbidity, duration of diabetes mellitus, hyperglycemic crisis, insulin therapy and HbA1c level, *IHD* Ischemic heart disease, *CI* confidence interval

## Discussion

Our present nation-wide survey showed the prevalence of IHD among Thai patients with diabetes mellitus was 3.54%. IHD was significantly associated with being male, age, being in Health Region 4, hypertensive comorbidity, presenting hyperglycemic crisis and insulin therapy. To our knowledge, this is the first report on the prevalence and risk factors of IHD among Thai patients with diabetes. The prevalence of IHD was 0.99% among the general population in Thailand reported in 1991 [11]. A related study in Sweden found a much higher prevalence of IHD (21.97%) among patients with diabetes including those aged from 45 to 74 years [13]. In contrast, our study enrolled all patients with diabetes aged from 35 to 97 years. However, when the same age groups as the Swedish study were analyzed, the prevalence of IHD was 2.75%. In the general population, the prevalence of IHD in Caucasian populations is normally higher than that among Asians [1, 7] and diabetes amplifies this morbidity. The present study enrolled patients with diabetes who received medical treatment in MoPH hospitals all over Thailand and public and private clinics in Bangkok under the NHSO. However, these populations did not include patients with diabetes who received medical treatment in health promoting hospitals (HPHs). These HPHs, primary care units of a community hospital, usually provide health care and medication for uncomplicated diabetes cases. The prevalence of IHD in this study may have been overestimated because the diabetic cases in HPHs were not included.

After adjusting for confounding factors by multivariate analysis, only older age, being male, being in Health Region 4, hypertensive comorbidity, presenting hyperglycemic crisis and insulin therapy remained significantly associated with IHD among patients with diabetes. Prevalence of IHD tended to be higher with older age similar to related studies in Sweden [13] and Finland [14]. Among the elderly, changes occurring in endothelial function include loss of arterial elasticity and reduced arterial compliance, so more vascular aging and degenerative processes lead to atherosclerosis disease [15]. In addition, the endothelium experiences changed arterial function by decreased nitric oxide and increased endothelin causing a pro-coagulant state and promoting vascular smooth muscle growth and exaggerated increased risk of cardiovascular events [16]. Another reason is the elderly often have a low frequency of physical activity. A recent study showed that adults, who were physically active at least twice weekly, had decreased risk of CHD [17, 18].

The result of our study was similar to studies in the US and in western and Asian populations [2, 9, 19–21]. Most studies have found that IHD was more common among males than females [9, 11, 14, 19, 20, 22]. IHD being common among males was probably related to hormonal effects. In the premenopausal period, females’ sex-hormones such as estrogen promote increased HDL and decreased LDL causing a cardio-protective effect [23, 24]. However, a related study in Pakistan showed that central adiposity is significantly higher among females compared with males regarding patients with type 2 diabetes mellitus [25]. Consequently, adiposity is positively related to IHD especially among females [26]. Patients presenting more adiposity may lead to IHD by adipocytokines by the inflammatory pathway causing vascular pathology and establishing atherothrombosis [27]. In addition, differing lifestyle or behavioral patterns between male and female indicate males present higher behavioral risks, e.g., from smoking, that predispose cardiovascular events [28].

However, several related studies have shown that higher BMI leads to risk for IHD [29–31]. The results of statistical analysis in this study found that a higher BMI exhibited a trend of dose response effect to IHD, but without significance. One result from the homogeneity in these diabetes populations is being categorized as overweight to obesity. In addition, patients, who have undergone a long duration with diabetes, may lead to presenting higher BMI. These diabetic populations are at greater risk for IHD; however, the duration of diabetes was not significant in the final model of this study.

The present study found that patients with diabetes, who present hypertensive comorbidity, are at increased risk for IHD. Hypertension is related to IHD and can be described by pathophysiology. The neuro-mediators of hypertension including angiotensin II can promote plasminogen activator inhibitor (PAI-1) expression and increased PAI-1 levels inhibiting the function of tissue plasminogen activator (tPA) resulting in increased myocardial infraction. In addition, the hypertensive stage adds extra pressure that can damage the arterial wall making it more vulnerable and building up plaque associated with atherosclerosis [32]. This study showed that hyperglycemic crisis was related to a high prevalence of IHD with an adjusted ORs of 1.53 (95% CI, 1.14–2.06). Acute hyperglycemia can attenuate endothelial function and reduce nitric oxide (NO) bioavailability [33, 34]. These actions promote monocyte and vascular smooth muscle cell migration into the intima and form macrophage foam cells, characterizing the initial morphological changes of atherosclerosis [35]. The study showed that insulin therapy was related to a high prevalence of IHD with an adjusted ORs of 1.40 (95% CI, 1.17–1.66). Insulin is more likely to be used among patients with more severe diabetes mellitus of longer duration and more complications such as chronic kidney disease. Therefore, these patients with insulin therapy have an increased number of cardiovascular events.

We have an explanation why IHD related to Health Region 4 in our study. The study showed this region had the highest prevalence of IHD at 6.4%. Health Region 4 is in central Thailand, a peripheral area near the Bangkok Metropolitan Area, where sufficient public health services are much more available. As a result, more patients can access services creating a higher load of reported cases. In addition, the area, which features several agriculture businesses, has more than enough dietary products combined with inappropriate patient dietary behaviors might have promoted a higher risk of cardiovascular disease. Consequently, the patients with diabetes in this area exhibited higher BMIs on average than others area, resulting in a higher risk for IHD [29, 30]. In addition, the patients with diabetic in Health Region 4 had higher age levels than the patients with diabetes in other areas. Although we adjusted age in the final model, the residual effect of age remained.

The study employed a cross-sectional design, and as such, the results could show only factors associated with IHD. The data presented in this study were obtained in the 2012–2013 Thailand DM/HT study of the NHSO from the Medical Research Network of the Consortium of Thai Medical Schools (MedResNet) central data management system. We were aware of missing data from this observational study. Even though this represented a relatively large sample size of the study population and some data were missing as from the nationwide observational (real life situation) study, the associations between factors and outcomes were able to be presented. In the study, we relied on evidence from medical records to identify ischemic heart disease; therefore, a small proportion of individuals with undiagnosed ischemic heart disease may have been misclassified non-differentially. The effect of misclassification decreases the observed effect size (odds ratio) of the associations (toward null). However, the officers who enter data in each hospital received proper training and verified reviewed medical records. The strength of this study was its nation-wide scope for IHD in a diabetic population. Thus, the finding of the study can be generalized and applied in others diabetic population.

## Conclusion

The prevalence of IHD in a diabetic population in this study was 3.54%, higher than the prevalence of IHD in the general population in most related reports. Factors associated with IHD included age, being male, hypertensive comorbidity, being in Health Region 4, presenting hyperglycemic crisis and insulin therapy. Our data emphasized that IHD was a problem among patients with diabetes. Diabetic patients should be regularly assessed for IHD and their risk factors should be better controlled. Moreover, the Ministry of Public Health managers and clinicians should provide further preventative strategies to attenuate cardiovascular disease.

## Additional file

Additional file 1: Univariate analysis of factor associated with ischemic heart disease in diabetic patients. (XLSX 18.7 kb)

## Abbreviations

BMI: body mass index
CI: confident interval
DBP: diastolic blood pressure
FPG: fasting plasma glucose
GFR_EPI: glomerular infiltration rate calculated by epidemiology collaboration formula
Hb: hemoglobin,
HbA1c: hemoglobin A1c
Hct: hematocrit
HDL: high density lipoprotein cholesterol
HT: hypertension
IHD: ischemic heart disease
LDL: low density lipoprotein cholesterol
LVH: left ventricular hypertrophy
MoPH: Ministry of Public Health
NHSO: National Health Security Office
SBP: systolic blood pressure
SD: standard deviation
TC: total cholesterol
TG: triglyceride
